# More than two components: complexities in bacterial phosphosignaling

**DOI:** 10.1128/msystems.00289-24

**Published:** 2024-04-09

**Authors:** Andrew Frando, Christoph Grundner

**Affiliations:** 1 Center for Global Infectious Disease Research, Seattle Children’s Research Institute, Seattle, Washington, USA; 2 Department of Pediatrics, University of Washington, Seattle, Washington, USA; 3 Department of Global Health, University of Washington, Seattle, Washington, USA; NIAID, NIH, Bethesda, Maryland, USA

**Keywords:** phosphosignaling, bacteria, kinases, phosphoproteomics

## Abstract

For over 40 years, the two-component systems (TCSs) have taken front and center in our thinking about the signaling mechanisms by which bacteria sense and respond to their environment. In contrast, phosphorylation on Ser/Thr and Tyr (*O*-phosphorylation) was long thought to be mostly restricted to eukaryotes and a somewhat accessory signaling mechanism in bacteria. Several recent studies exploring systems aspects of bacterial *O*-phosphorylation, however, now show that it is in fact pervasive, with some bacterial proteomes as highly phosphorylated as those of eukaryotes. Labile, non-canonical protein phosphorylation sites on Asp, Arg, and His are now also being identified in large numbers in bacteria and first cellular functions are discovered. Other phosphomodifications on Cys, Glu, and Lys remain largely unexplored. The surprising breadth and complexity of bacterial phosphosignaling reveals a vast signaling capacity, the full scope of which we may only now be beginning to understand but whose functions are likely to affect all aspects of bacterial physiology and pathogenesis.

## THE EVOLVING VIEW OF BACTERIAL PROTEIN PHOSPHORYLATION

**Fig 1:**
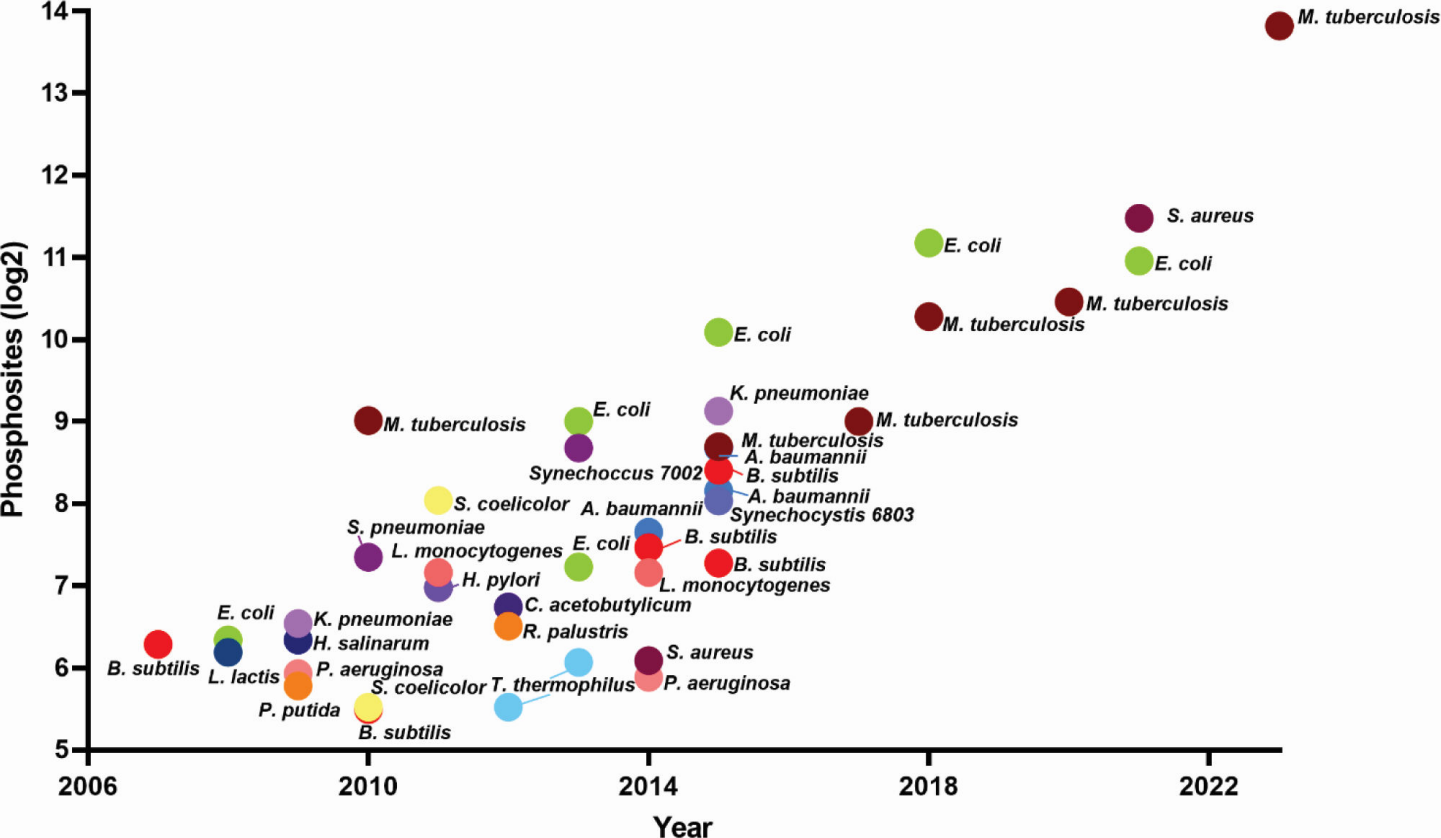
The rapid rise in identifications of *O*-phosphorylation sites in bacteria. The number of unique phosphosites from a selection of MS-based phosphoproteomic studies are shown. Significance metrics for site identification were adopted from the original studies and may vary between studies.

Protein phosphorylation on a single site can alter the fate of the entire cell. The efficiency and versatility of this modification is the basis for cellular signal transduction in prokaryotes and eukaryotes, but both evolved idiosyncrasies in the ways in which phosphate is added, removed, and detected (1, 2). The chemistries underlying protein phosphorylation in cells fall broadly into two categories: stable phosphate bonds (pSer, pThr, and pTyr, or *O*-phosphorylation) and labile phosphate bonds (pAsp, pArg, and pHis, also pCys, pLys, and pGlu) (3). The bacterial two-component systems (TCSs) with their pHis/pAsp transfer from histidine kinase to response regulator were long considered the canonical mechanism by which bacteria sense and respond (4). *O*-Phosphorylation, in contrast, was initially thought to be an exclusively eukaryotic signaling mechanism, and its identification in bacteria lagged behind that in eukaryotes by almost a quarter century (with the exception of a protein kinase activity described in *Escherichia coli* as early as 1969 [5]). The initial phosphoproteins, kinases, and functions for bacterial *O*-phosphorylation were identified in a series of seminal studies (for excellent timelines, see Dworkin [6] and Mijakovic and Macek [7]). Together with the increasing number of Hank's-type Ser/Thr kinases identified in the rapidly growing number of bacterial genome sequences, these studies began to challenge the notion that bacterial *O*-phosphorylation was mostly an accessory signaling mechanism in bacteria. The arguably first global analysis of bacterial *O*-phosphorylation predated the wide use of mass spectrometry (MS) for phosphoprotein detection but already provided remarkable detail of *O*-phosphorylation in *E. coli* (8). Using radioactive labeling with ^32^P and two-dimensional gel electrophoresis, the study showed 128 phosphoproteins, shifts in the phosphorylation pattern in response to different growth conditions, and a bias for pSer in the *E. coli* phosphoproteome. The marriage between phosphopeptide enrichment through immobilized metal-affinity chromatography and MS in the early 2000s then led to the emergence of global phosphoprofiling (9). In eukaryotes, thousands of phosphosites are now routinely identified, and in yeast, mice, and humans, as many as 75% of all proteins are simultaneously phosphorylated (10). Similar studies to identify global *O*-phosphorylation in bacteria have yielded fewer sites in the past, for example, in the first MS-based bacterial phosphoproteomic studies of *Bacillus subtilis* (11), *Campylobacter jejuni* (12), and *E. coli* (13). Even with the smaller proteomes of bacteria, the numbers of phosphosites implied that no more than a few percent of bacterial proteins were phosphorylated. While these first bacterial phosphoproteomes were conceptually important, they seemed to support the prevailing idea that bacterial *O*-phosphorylation is less common and less consequential than in eukaryotes. Several recent studies are beginning to revise this view. The number of phosphosites identified in MS-based studies is climbing sharply, with several recent studies showing pervasive *O*-phosphorylation (14–22) (Fig. 1). In fact, in *Mycobacterium tuberculosis* (*Mtb*), a degree of phosphorylation that is on par with *O*-phosphorylation in eukaryotes has recently been identified (14) (Fig. 1). What these numbers suggest is that we are probably still far from a comprehensive inventory of *O*-phosphorylation for most bacteria.

**Fig 2:**
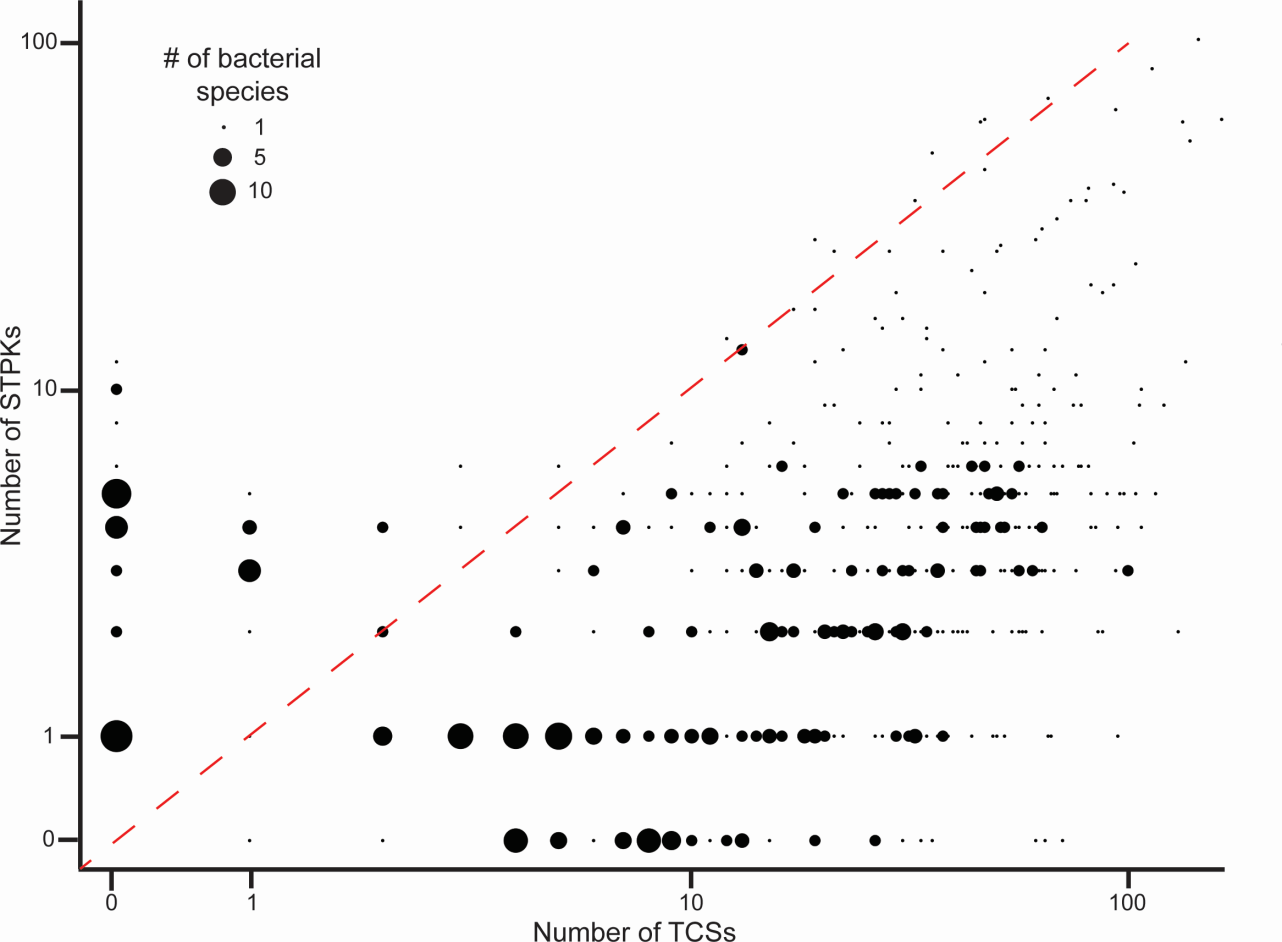
The relationship between the number of HKs and STPKs in 541 bacterial genomes. The numbers of STPKs from 541 bacterial species were plotted against the number of TCSs as identified in reference 23. A total of 42 bacterial species code for STPKs but not HKs, and 51 code for HKs but not STPKs. Most bacteria (>400) have more HKs than STPKs, and 26 have more STPKs than TCSs. *Sorangium cellulosum* with 149 TCSs and 323 STPKs was excluded to avoid scale contraction. Diagonal represents a 1:1 ratio.

The relative significance of *O*-phosphorylation versus TCSs for bacterial signaling probably varies by bacterial species. A census of the respective kinases in bacteria indeed shows a wide range of the number and ratio of TCSs and STPKs. A compilation of prokaryotic signal transduction systems from over 500 bacterial genomes (23) identified 16,000 histidine kinases of TCSs and over 3,000 STPKs, or on average 4.7 HKs per STPK. Interestingly, there is no apparent correlation between the number of HKs and STPKs encoded by individual organisms (Fig. 2), as one might expect if there was either coordinated evolution of the two systems (positive correlation) or a functional tradeoff between HKs and STPKs (negative correlation). While most bacterial species surveyed coded for more HKs than STPKs (>400), about 10% of the surveyed species only had one or the other. The lack of a clear correlation might suggest that the two phosphosystems evolved largely independently. Interestingly, examples of STPKs controlling TCS activity are increasingly reported (24–32), both on the level of the His kinase and the response regulator. Evidence for regulation the other way around, however, is scant, suggesting a signaling hierarchy in which the STPKs control many TCSs (32) and fine-tune and modulate their cellular outputs.

## LABILE PROTEIN PHOSPHORYLATION

**Fig 3:**
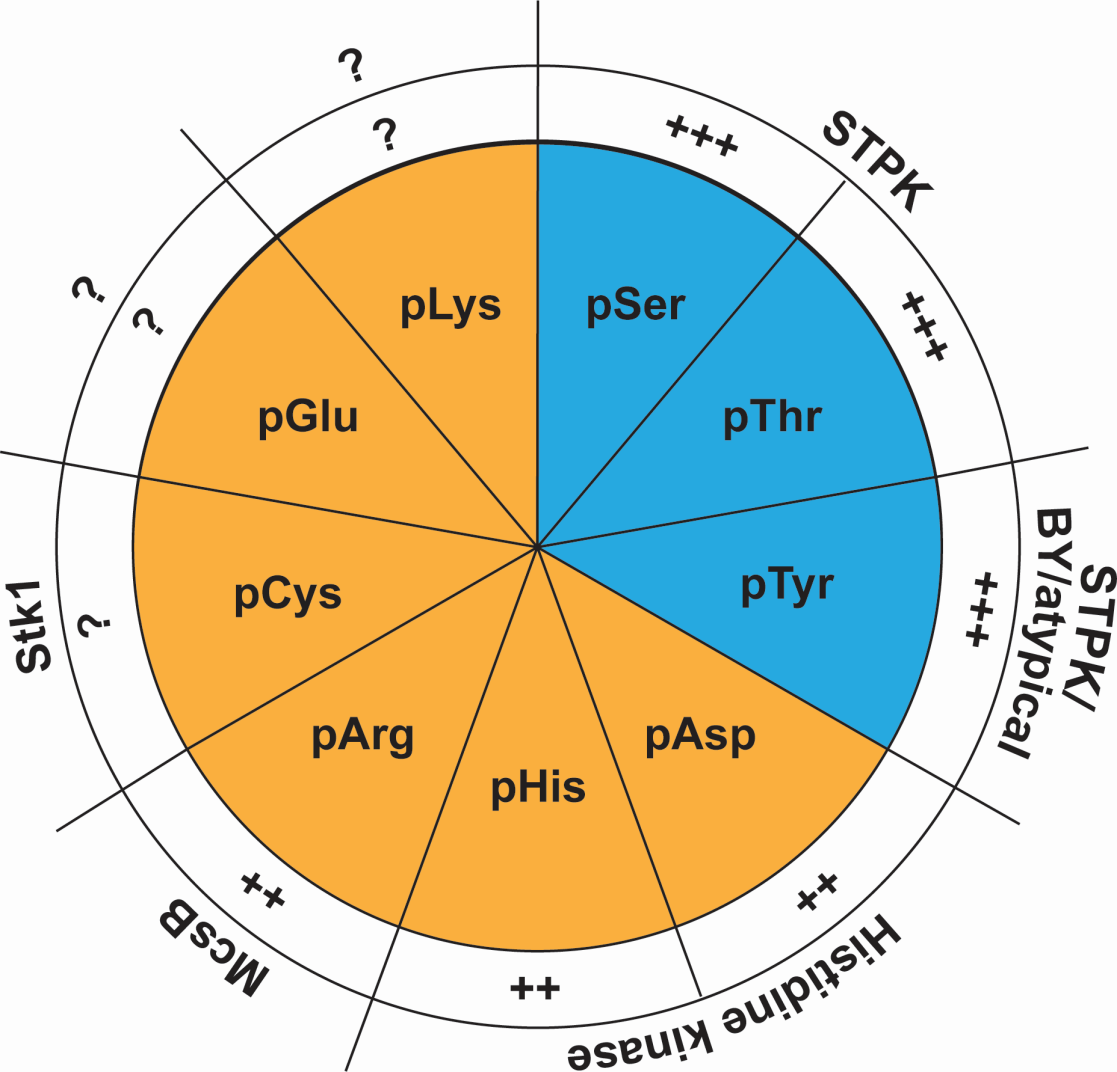
The spectrum of bacterial protein phosphorylation. The nine amino acids that can be phosphorylated in proteins, their prevalence in bacteria, and the kinases known to phosphorylate these residues are shown. Blue: chemically stable phosphates. Orange: chemically labile phosphates.

Of the nine amino acids that can be modified by phosphate in proteins, only three are chemically stable (Fig. 3). The other six are labile, resulting in short-lived phosphorylation and loss of phosphate in standard MS sample preparation and peptide fragmentation protocols. These labile protein phosphoamino acids are pAsp, pArg, pCys, pHis, pLys, and pGlu, and their identification has been exceedingly challenging (for an excellent review of the chemistry of labile phosphates, see reference 3). With a combination of tailored sample preparation protocols, chemical modification, and MS protocols using optimized fragmentation techniques (17–19, 33, 34), the analysis of labile phosphoproteins and phosphoproteomes is now becoming technically more feasible. These technical advances have already transformed the study of labile phosphates and revealed a remarkable additional complexity in bacterial protein phosphorylation by labile phosphates.

Protein Arg phosphorylation is arguably the best understood labile phosphomodification. The phosphoramidate (P-N) bonds of pArg (35), similar to pHis, are short-lived in the cell and unstable in conventional MS workflows. However, recent studies in *B. subtilis* showed considerable protein pArg modification, with >200 sites detected in an Arg phosphatase mutant strain (36). A landmark study also showed a central functional role of bacterial pArg: in *B. subtilis*, pArg serves as a degradation tag for protein turnover by the Clp protease similar to the way ubiquitin functions as a degradation tag for the proteasome (37). Also in *B. subtilis*, Arg phosphorylation was found to be critical for spore germination. In this case, dephosphorylation of multiple pArg by the Arg phosphatase YwlE controlled germination (38). The critical function of a pArg phosphatase is particularly striking as labile phosphates are more transient and their dephosphorylation generally thought to be less tightly controlled than that of *O*-phosphorylation sites. A chemoproteomic approach using chemical traps to stabilize the equally unstable pAsp recently identified pAsp on *E. coli* (39), *B. subtilis*, and *Pseudomonas aeruginosa* (40) response regulators, consistent with biochemical studies over the last 40 years showing that response regulators are phosphorylated on Asp. Remarkably, these studies also identified many pAsp sites on proteins other than response regulators (100, 141, and 198 in *E. coli*, *B. subtilis*, and *P. aeruginosa*, respectively [39, 40]). In the case of *P. aeruginosa*, the sequences around the pAsp site showed modest similarity with the pAsp motif also found in response regulators, suggesting that they might also receive their phosphate from His kinases. These MS-based, global studies are welcome progress from the previous approaches to studying labile phosphate that almost exclusively relied on [γ−^32^P]ATP-labeling of one recombinant protein at a time. Although the first protein phosphorylated on His was identified in eukaryotes (41), proteome-wide detection of pHis originated in bacteria. Through optimization of MS conditions that preserve pHis, Potel et al. identified a remarkably large His phosphoproteome of 246 sites in *E. coli:* ~10% of all phosphorylation sites (18). Subsequent studies in human cell lines then also detected upwards of 500 pHis sites (42, 43), and a database dedicated to the curation of pHis sites, HisPhosSite, was established (44).

The study of pCys, pGlu, and pLys has met more challenges, and their prevalence and functions remain poorly defined in bacteria (and eukaryotes). Evidence for the three is anecdotal, and functional characterization is largely lacking. The perhaps only known exceptions are the Cys phosphorylation of the glucose transporter of *E. coli* (45) and that of the SarA/MgrA family proteins SarA, SarZ, and MgrA in *Staphylococcus aureus*, which affects their transcriptional regulatory effects (46). Several of the known modifications on pCys, pGlu, and pLys also raise a broader question about protein phosphorylation by labile phosphates: when is phosphorylation a signaling event and when is it merely an intermediate state in enzyme catalysis? One example of the latter is the transient pCys intermediate in Tyr phosphatases that is part of the dephosphorylating mechanism (47). Yet another example of kinase-independent protein phosphorylation is that of the phosphoglucosamine mutase, GlmM. An essential enzyme for the synthesis of a cell wall precursor, GlmM requires phosphorylation on a Ser residue for activity. Rather than an additional kinase phosphorylating GlmM, however, GlmM has autophosphorylating activity (48). Whether this phosphate is then transferred to the substrate is unclear, but the general phosphorylation-dependent activation is also a feature of GlmM in other bacteria, yeast, and rabbit, and perhaps even in the entire hexosephosphate mutase family. These examples show that the line between phosphorylation that functions in true signaling events and that of enzyme intermediates can be blurred. First phosphoproteomic analyses of pCys, pGlu, and pLys have been accomplished in eukaryotes (34), but are lacking in bacteria, such that their prevalence remains unknown.

Adding to the challenges of understanding labile phosphorylation is that the responsible phosphoenzymes are mostly unknown. One exception is the well-characterized *B. subtilis* Arg kinase McsB (49), which has orthologs in many bacterial species including Gram-negatives, suggesting that it could be a general bacterial Arg kinase. Except for the TCS's His kinases, bacterial His kinases are similarly unknown, and so are dedicated Asp kinases (Fig. 3). In this context, it is useful to remember that the His kinases of TCSs only autophosphorylate on His but transfer phosphate *in trans* to Asp. Although pHis could in principle be a phosphodonor for another His (50), such activity has not been described, so that a true His kinase in the typical sense also remains undefined. The identification of enzymes responsible for labile phosphorylation and dephosphorylation will be very useful as their genetic perturbation is often a direct way to understand the modification they cause, and deletion of phosphatases has helped increase the size of the labile phosphoproteome that can be detected, for example, for pArg (19). Bacterial Tyr phosphorylation is mediated by a number of unusual kinases such as BY tyrosine kinases and atypical Tyr kinases (51–53). It appears plausible that bacteria also mediate labile phosphorylation by unique kinases. Since these kinases likely lack distinctive sequence or structural features, their identification will require innovative tools. Several activity-based methods that are agnostic to sequence and structure have recently emerged for the identification of such outlier enzymes in large and complex proteomes by unbiased methods (54). Alternatively, some of the canonical *O*-phosphorylation enzymes could also phosphorylate other residues, analogous to how some Ser/Thr kinases function as dual-specificity kinases that also phosphorylate bacterial Tyr (55) or Cys (46) residues. New ideas will be needed along with new technologies to explore these more elusive labile phosphorylation events, its associated enzymes, and ultimately functions.

## BACTERIAL PHOSPHOSIGNALING NETWORKS

The TCSs are generally characterized by a linear pathway structure and minimal crosstalk (4), although recent data suggest more complicated signaling pathway architectures for TCSs in some bacteria (56, 57). In contrast, STPK-associated signal transduction networks in eukaryotes resemble scale-free networks with extensive interconnectivity (58), with sometimes hundreds of cellular substrates that crosstalk with many signaling pathways. Where on this spectrum from linear pathway to signaling network do bacterial *O*-phosphorylation systems fall? The first system-level characterizations to dissect the kinome and its wiring in prokaryotes were carried out in *Mtb*. Although *Mtb* is not a model organism by any means, its 11 STPKs are among the best characterized, not least because of their promise as therapeutic targets. An *Mtb* STPK was also the first to be characterized structurally, in work that revealed the close structural similarity between prokaryotic and eukaryotic STPKs (59, 60). The first large phosphoproteomic study of *Mtb* and the largest of its kind in bacteria at the time identified >500 phosphosites on >300 proteins, a number that suggested pervasive regulation of *Mtb* cellular processes by STPKs (61).

Initial mapping of STPKs to peptide substrates identified substrate selectivity and the first kinase-substrate relationships for specific proteins (61). The *in vitro* essential *Mtb* STPKs PknA and PknB were thereafter the focus of several systems-level profiling studies, with studies probing the effects of genetic and chemical inhibition on the phosphoproteome, transcriptome, and metabolome (24, 62). These studies showed a large regulatory footprint of PknA and PknB and an overlapping activity that was presaged by their adjoining genomic location. These studies of *Mtb* began to reveal the pleiotropic and more combinatorially connected nature of these STPKs, suggesting that *Mtb* STPKs might work together in a larger, more interconnected network reminiscent of eukaryotic STPK networks. Such a phosphorylation network was indeed recently shown in yet another study of *Mtb* (14). This study combined STPK deletion and overexpression of all but one of the eleven STPKs with MS analysis of the resulting phosphorylation changes and assigned kinases to substrates across the kinome. This work showed an expansive, distributed, and coordinated phosphosignaling system that is similar in complexity to the most complex eukaryotic STPK signaling networks—a complexity that has not previously been seen in bacteria. The specific features of the network include a high degree of apparent redundancy of substrate phosphorylation: more than two thirds of phosphoproteins were phosphorylated by more than one STPK, and some proteins were phosphorylated by all ten tested STPKs. This redundancy might provide an explanation to the puzzling observation that, despite large phosphorylation and transcriptional effects of these STPKs, knockout phenotypes of all but the two *in vitro* essential STPK mutant strains in culture were subtle or absent (14, 63). In a network context, such an absence of phenotypes can be interpreted as functional redundancy that confers robustness to a system, similar to that seen in other signaling and transcriptional networks (64). Notably, previous studies have primarily probed STPK loss-of-function mutations or their chemical inhibition. This approach is premised on the basal activity of the STPK under the condition tested, which is not always a correct assumption. In fact, several of the *Mtb* STPKs did not have basal expression in standard broth culture, which rendered kinase knockouts' effect on the phosphoproteome mute, and required overexpression for their activation (14). Other bacterial STPKs are also in a similarly inactive basal state and might also require activation to reveal cellular substrates that are not detectable by gene deletion approaches. For example, an overexpression approach was also needed to identify substrates of the *E. coli* HipA family Ser/Thr kinase HipH (65). A striking observation from a kinome-phosphoproteome study in yeast was that almost half of phosphosite changes upon kinase depletion behaved in the opposite direction as expected, that is, more abundant phosphorylation when a kinase was deleted (66). This counterintuitive behavior can be explained by indirect STPK action, such as a kinase phosphorylating and inhibiting another or by activating a phosphatase. In this way, these opposite-sign responses to kinase deletion can indirectly gauge the interconnectedness of the STPKs themselves. In *Mtb*, 8% of phosphorylation events had the opposite directionality, suggesting some but a lower degree of connectedness than in yeast (14).

## THE PHOSPHORYLATION FUNCTION CONUNDRUM

A central challenge facing the phosphosignaling field in general also presents itself in bacteria as we encounter ever larger phosphoproteomes—to distinguish phosphorylation events that are functional from those that are not. The lack of tools to differentiate functional from non-functional phosphorylation events, either by prediction or experiment, on a scale anywhere near the thousands of sites now routinely detected makes this problem especially daunting. By one estimate, as many as half of all phosphorylation sites are non-functional (67), but this hypothesis remained untested. The conventional standard for validating regulatory phosphosites is phosphosite mutation followed by a phenotypic assay. Identifying function of a phosphosite in this way requires *a priori* knowledge of the phosphoprotein's function, assays specific for that function, and individual phosphosite mutation. While Ala and Asp/Glu can ablate or mimic pSer and pThr (68), respectively, pTyr lacks alternative amino acid proxies for mimicking the phosphorylated state, and mutant synthesis can be labor-intensive. Additionally, in many bacteria, over a third of proteins have no annotated function whatsoever (69) to test for by biochemical assays, together making this approach incompatible with high-throughput characterization.

Recent work has begun to experimentally test for phosphosite function on a larger scale by embracing a brute-force mutation and phenotyping approach. These studies provide fascinating insights into the question of phosphosite function and predict that, in fact, many if not most sites have a function when probed deeply enough. In the most comprehensive such study to date, the effect of ablating Ser/Thr and Tyr phosphosites on the growth of yeast was studied in >100 different growth media (70). The study found that single mutation of >40% of 474 phosphosites with diverse characteristics leads to growth phenotypes. In another study, a metabolomic readout was used to assess the effects of phosphosite mutation on glycolysis and related enzymes in *E. coli* (71). Using this sensitive readout in combination with growth assays, the study revealed that >76% (44/52) of phosphosites had metabolic consequences. These estimates are probably on the low end since phosphomimetic mutation and combinatorial phosphorylation were not tested, and even the >100 media conditions of the yeast study likely missed some relevant environmental contexts. Although it remains untested how well these findings transfer to other bacteria or labile phosphorylation sites, the results suggest that spurious phosphorylation is the exception, and that large numbers of functional phosphosites tune bacterial physiology in a highly context-dependent way.

Another important aspect of phosphosite function is stoichiometry. The stoichiometry, or occupancy, of a phosphosite is likely related to its function such that high occupancy might predict function. The precise global measurement of occupancy, however, remains a major challenge, requires measuring phosphorylated and non-phosphorylated versions of the same peptide, and typically provides only relative quantitation. Some approaches provide reliable occupancy measures but require labeled peptide standards and targeted MS detection that do not yet lend themselves to proteome-wide analysis (i.e., Hahn et al. [72]). Thus, it is not surprising that most phosphoproteomic data sets currently provide very limited and only semiquantitative information on phosphosite occupancy. Yet, two observations indicate that the occupancy of phosphosites in bacteria is in general lower than in eukaryotes: the number of phosphopeptides detected in bacteria is consistently lower than in eukaryotes, despite over 20 years of global, proteome-wide studies. The second is the striking difference in the overall concentration of protein-linked phosphate in prokaryotes and eukaryotes. The concentrations of these have been directly measured in at least one case: the mouse proteome contained approximately eighty times as many phosphates per mole of protein than the *Corynebacterium glutamicum* proteome (73). Although high occupancy might predict phosphosite function, low occupancy does not necessarily rule it out but might reflect localized or temporal phosphorylation of a subpopulation of protein that is nonetheless functional. Additional unresolved questions are the implications of combinatorial phosphorylation that has not yet been explored in any depth. As is true for eukaryotes, extensive intersections between different types of PTMs are now also increasingly apparent in bacteria (74) and likely add further layers of complexity to bacterial signal transduction.

The idiosyncrasies of bacterial phosphosignaling are likely to provide new insights into bacterial signal transduction, and in particular the labile phosphates are understudied still. Phosphosignaling, at least in some bacteria, appears to be no less complicated than in eukaryotes, and it is all but certain to hold answers to many questions about bacterial physiology and pathogenicity.
